# Role of Tryptophan in Microbiota-Induced Depressive-Like Behavior: Evidence From Tryptophan Depletion Study

**DOI:** 10.3389/fnbeh.2019.00123

**Published:** 2019-06-04

**Authors:** Iva Lukić, Dmitriy Getselter, Omry Koren, Evan Elliott

**Affiliations:** ^1^Molecular and Behavioral Neuroscience, Bar-Ilan University, Safed, Israel; ^2^Microbiome Research Laboratories, The Azrieli Faculty of Medicine, Bar-Ilan University, Safed, Israel

**Keywords:** tryptophan, serotonin, microbiota, brain, depression

## Abstract

During the past decade, there has been a substantial rise in the knowledge about the effects of gut microbiota on host physiology and behavior, including depressive behavior. Initial studies determined that gut microbiota can regulate host tryptophan levels, which is a main serotonin precursor. A dysfunctional serotonergic system is considered to be one of the main factors contributing to the development of depression. Therefore, we hypothesized that regulation of brain tryptophan and serotonin can explain, at least partly, the effects of microbiota on depressive behavior. To test this hypothesis, we examined depressive-like behavior and brain levels of serotonin and tryptophan, of germ free (GF) and specific-pathogen free (SPF) mice under basal conditions, or after acute tryptophan depletion (ATD) procedure, which is a method to decrease tryptophan and serotonin levels in the brain. In basal conditions, GF mice exhibited less depressive-like behavior in sucrose preference, tail-suspension and forced swim tests, compared to SPF mice. In addition, in mice that were not subjected to ATD, GF mice displayed higher levels of tryptophan, serotonin and 5-hydroxyindoleacetic acid (the main degradation product of serotonin) in medial prefrontal cortex (mPFC) and hippocampus (HIPPO), compared to SPF mice. Interestingly, ATD increased depressive-like behavior of GF, but not of SPF mice. These behavioral changes were accompanied by a stronger reduction of tryptophan, serotonin and 5-hydroxyindoleacetic acid in mPFC and HIPPO in GF mice after ATD, when compared to SPF mice. Therefore, the serotonergic system of GF mice is more vulnerable to the acute challenge of tryptophan reduction, and GF mice after tryptophan reduction behave more similarly to SPF mice. These data provide functional evidence that microbiota affects depression-like behavior through influencing brain tryptophan accessibility and the serotonergic system.

## Introduction

Depression is one of the most common psychiatric conditions, with lifetime prevalence ranging from 6 to 18% across different populations worldwide (Kessler and Bromet, 2013). Most often it is a recurrent condition, which leads to reduced quality of life in addition to an increased risk for other medical problems, such as diabetes, heart disease, and stroke (Merikangas et al., 2007; Kessler et al., 2009; Bhattacharya et al., 2014). All these factors make depression one of the leading contributors to global chronic disease burden.

The serotonin hypothesis of depression is among the first suggested biological causes of this disorder, dating from about 50 years ago, that was based on unexpected findings regarding several drug actions (Coppen, 1967; Albert et al., 2012). Although the understanding of the complexity of brain function and etiology of depression has increased much in the past 50 years, a dysfunctional serotonergic system is still considered to be one of the main factors contributing the development of depressive symptoms (Belmaker and Agam, 2008; Albert et al., 2012; Fakhoury, 2016).

In the brain, serotonin or 5-hydroxytryptamine (5-HT) is synthetized in serotonergic neurons originating from raphe nuclei, from amino acid tryptophan (TRP; Ruddick et al., 2006; Lesch and Waider, 2012). TRP crosses blood brain barrier *via* a specific transporter, competing with other large neutral amino acids to enter the brain (Ruddick et al., 2006). The first enzyme in brain serotonin synthesis pathway is tryptophan hydroxylase 2 (TPH2; Walther et al., 2003) that is only half-saturated by normal physiological TRP concentrations, and represents a rate-limiting step of the pathway (Boadle-Biber, 1993). After the release of 5-HT, the neurotransmitter is reuptaken by a serotonin transporter (SERT), to the presynaptic terminal (Borowsky and Hoffman, 1995) and can be further metabolized to 5-hydroxyindoleacetic acid (5-HIAA), as a final product of its degradation (Jacobs and Azmitia, 1992).

Certain genetic variants of serotonin receptors (Lemonde et al., 2003), SERT (Hoefgen et al., 2005; Caspi et al., 2010), as well as TPH2 (Zill et al., 2004; Zhang et al., 2005) were associated with a higher risk of depression. In addition, decreased levels of TRP in blood (Cowen et al., 1989) and 5-HIAA in cerebrospinal fluid were reported in some depressed patients (Ashcroft et al., 1966; Dencker et al., 1966). However, some of the most consistent findings regarding the role of 5-HT in the pathophysiology of depression come from studies of acute tryptophan depletion (ATD). The procedure involves dietary manipulation in order to decrease TRP levels, and subsequently brain 5-HT synthesis. TRP depletion leads to acute mood lowering effects in patients with depression in remission, as well as in first degree relatives of depressed patents but not in healthy subjects without family history of depression (Booij et al., 2003; Ruhé et al., 2007). These data suggest that vulnerability of the serotonin system is implicated in the development of depressive episode (Jans et al., 2007).

During the past decade, there was a huge rise in knowledge about the effects of gut microbiota on host physiology and behavior, including depressive behavior. For example, germ free (GF) mice exhibit less behavioral despair compared to their conventional counterparts (De Palma et al., 2015; Campos et al., 2016). Also, patients with depression have altered gut microbiota compared to healthy subjects (Jiang et al., 2015; Kelly et al., 2016; Zheng et al., 2016).

One of the potential mechanisms through which gut microbiota could impact depressive behavior is through influence on TRP and brain 5-HT metabolism. TRP, which is an essential amino acid, is mainly supplied from ingested food and is a biosynthetic precursor of a large number of host as well as microbial metabolites (Milligan et al., 1978; Lee and Lee, 2010; Agus et al., 2018). In addition, studies have shown that gut microbiota directly regulates the blood TRP levels through stimulation of serotonin production of the gut enterochromaffin cells (Yano et al., 2015). Indeed, GF mice have higher plasma levels of TRP (Wikoff et al., 2009; Clarke et al., 2013), but lower blood levels of serotonin, compared to conventional mice. However, GF mice have higher 5-HT levels in the brain (Clarke et al., 2013). Further, some studies of gut microbiota alterations in depressed patients found increased levels of *Alistipes*, a bacterial genus that produce indole from TRP (Jiang et al., 2015). The authors suggested that its enrichment could potentially decrease TRP availability for host cells, thus impacting the balance of host brain serotonergic system. While these studies have given corroborating arguments that brain accessibility to TRP and its effect on serotonin production may partially explain the effects of microbiome on depressive-like behavior, there is yet little direct functional evidence to make this mechanistic claim.

In this study, we hypothesize that microbial influence on the availability of TRP to the brain may be a mechanism explaining, at least partly, the effects of the microbiome on depressive-like behavior. To gain evidence for this hypothesis, we performed TRP depletion in GF mice and specific-pathogen free (SPF) mice, to test if TRP reduction would attenuate the anti-depressive behavior of GF animals. Indeed, we determined that in basal conditions GF mice exhibit less depressive-like behavior compared to SPF mice. However, their serotonergic system is more vulnerable to the acute challenge of TRP reduction, leading to that GF mice act more similarly to SPF mice after TRP reduction. Therefore, these data provide direct functional evidence that microbial effects on depression-like behavior can be mediated through its effects on brain TRP accessibility and consequently serotonergic system.

## Materials and Methods

### Mice

All animals used in the study were bred and maintained in animal facilities at our research institute. SPF Swiss Webster male mice were housed in SPF vivarium with 12 h light/12 h dark cycle, at 22°C, with food and water available *ad libitum*. GF Swiss Webster male mice were housed in semi-solid GF isolators, also under a 12 h light/12 h dark cycle, at 22°C, with autoclaved food and water available *ad libitum*. Three to five mice were housed per cage and the experiment was carried out at 8–10 weeks of age. In the study of behavioral differences between GF and SPF animals, a total of 20 mice was used (10 mice per group). In the study of the effects of ATD on both GF and SPF animals, a total of 44 mice were used (11 mice per group). All experimental protocols were approved by the Animal Care and Use Committee of Bar-Ilan University.

### Behavioral Testing

Behavioral tests, except sucrose preference, were performed during the light phase of the diurnal cycle, between 12 a.m. and 16 p.m. On the day of behavioral testing, SPF as well as GF mice were brought to the behavioral room in the SPF facility, at least 1 h before the test, for acclimatization.

Sucrose preference test (SPT) was used to assess anhedonic behavior in mice. The test was carried out in home cages, while mice were single-housed by dividers. Each of the mouse was presented with two pipettes to choose freely: one containing drinking water and the other 0.5% sucrose solution. The position of pippets was switched every 24 h to eliminate side preference as a confounder. The test lasted 5 days, with the 1st day considered as habituation, while the next 4 days were considered as the test. During that time consumption of water and sucrose solution was measured and sucrose preference was calculated as a percentage of the volume of sucrose solution intake divided by the volume of total fluid intake.

Tail suspension test (TST) was performed by suspending mice by their tail for 6 mins, with the tape applied about 1 cm from the tip of their tail, under low light conditions (25 lux). Behavior other than trying vigorously to escape was scored as immobility, and considered as an indicator of depressive-like behavior (i.e., small movements with only front legs as well as swinging were not considered as mobility; Can et al., 2011b). The maze was cleaned with 10% alcohol after testing of each mouse. All testing was recorded by a video camera, and scoring was done manually.

Forced swim test (FST) was performed using modified procedure with pre-test 24 h before the test day (Dulawa et al., 2004). Both pre-test and test lasted 6 min and were conducted in plastic buckets 21 cm in diameter and filled with 24 ± 1°C water 15 cm from the bottom, under low light conditions (25 lux). After testing, mice were dried, and then returned to their home cages. Immobility, defined as the absence of active swimming with all four paws and tail moving, was scored during all 6 mins on the test day as a sign of depression-like behavior (Dulawa et al., 2004; Can et al., 2011a). All experiments were recorded by a video camera, and scoring was done manually.

### Experimental Procedures

#### Characterization of Behavioral and Gene Expression Differences Between SPF and GF Mice

After SPT was done, TST and FST were performed on the next three consequent days. In the case of GF mice, they were taken out from the isolators before TST test, and till the end of behavioral experiments, they were housed in the SPF facility.

Separate cohorts of GF and SPF mice were used for analyses of dorsal raphe nucleus (DRN) gene expression. GF mice used for these analyses were sacrificed 1 h after being taken out from the isolator.

#### Acute TRP Depletion in SPF and GF Mice

Prior to TRP depletion procedure, mice were handled for 3 days and once gavaged with protein-carbohydrate mixture with TRP, in order to habituate to the procedure. On the experimental day, mice were food deprived for 5 h in order to avoid the effects of TRP available in regular chow. After starvation, mice were gavaged with protein-carbohydrate mixture containing TRP (TRP^+^ group, 0.28% TRP of the total protein amount) or without TRP (TRP^−^ group). The composition of the given protein-carbohydrate mixture is shown in Table 1. Carbohydrates are added to the mixture in order to stimulate protein synthesis, leading to an optimal decrease of TRP levels relative to other large amino acids, which is the major factor determining TRP accessibility to the bran (Fernstrom and Wurtman, 1997; van Donkelaar et al., 2011). Mice were assigned to groups randomly and received two doses of 10 ml/kg of the corresponding mixtures, within 30 min interval. The behavioral testing or sacrifice was performed 2 h after application of the first dose. This treatment regime was chosen according to the previous study showing that it can efficiently reduce TRP levels in the blood, compared to all other large neutral amino acids, in Swiss Webster mice (van Donkelaar et al., 2010).

**Table 1:** Composition of protein-carbohydrate mixture and amino acid content in gelatine-based protein.

Substance	Amount in 10 ml water (g)
Protein	10
Alanin	1.10
Arginine	0.93
Aspartic acid	0.67
Cystein	0.01
Glutamic acid	1.14
Glycin	2.90
Histidin	0.10
Hydroxylysine	1.45
Hydroxiproline	0.12
Isoleucin	0.18
Leucine	0.34
Lysin	0.45
Methionin	0.10
Phenylalanin	0.26
Proline	1.76
Serine	0.38
Threonin	0.22
Tryptophan	0.00
Thyrosin	0.10
Valiun	0.33
Glucose	10
KCl	0.095
CaCl_2_*2H_2_O	2.32
L-Tryptophan (TRP^+^ group)	0.28
L-Tryptophan (TRP^−^ group)	0

The schedule of the treatment and subsequent tests and sacrifices was as follows. Food deprivation started at 7 a.m., the first dose of the corresponding mixtures was given at 12 p.m., and behavioral testing or euthanasia started at 14 p.m. First, TST was performed, and after 1 day of rest, FST was performed on the next two consecutive days. At the end of each testing day, mice were returned to their home cages and had access to the food *ad libitum*. All the procedures were done in behavioral room in the SPF animal facility. GF mice were taken out from the isolators 1 day before the 1st day of testing and placed in isocages. On the days of testing, they were brought to the behavioral room of the SPF facility, and after the tests, they were returned to isocages. Two days after completing behavioral experimentation, mice were euthanized and brains extracted.

### Brain Tissue Collection

Mice were rapidly decapitated and their brains were quickly removed. The medial prefrontal cortices (mPFC), the hippocampi (HIPPO) and dorsal rapeh nucleus (DRN) were isolated using brain matrix and gauge 13 (for mPFC and HIPPO) or 14 (for DRN). Brain tissues were immediately frozen on dry ice and stored at −80°C until further application.

### Neurochemical Analyses

After weighting, the desired tissue was homogenized with lysis buffer containing 1.8% perchloric acid and 10 mM ascorbic acid. After 15 min incubation on ice, the homogenates were centrifuged for 30 min 20,000x *g*, at 4°C and the supernatants were used for subsequent measurements according to Yamaguchi et al. (1981). The HPLC analyses were performed using C18 reversed-phase column (InfinityLab Poroshell 120 EC-C18, Agilent), and Agilent 1260 Infinity II system. The mobile phase was 10 mM potassium phosphate buffer with 5% methanol (pH 5.0) and the flow rate was 1 ml/min. TRP, 5-HT and 5-HIAA were identified by fluorescence detector, with wavelength set at 285 nm for excitation, and at 345 nm for emotion. During the HPLC procedure, all samples and standards were kept at 4°C and protected from light. The desired compounds were identified according to their retention times, and their amounts were calculated according to the standard curves prepared with different concentrations of the corresponding standards. The final tissue amounts of TRP, 5-HT and 5-HIAA were expressed in pg per mg of the tissue.

### Gene Expression Analyses

Total RNA was extracted by PureLink RNA Mini Kit (Invitrogen, Carlsbad, CA, USA) according to the manufacturer’s protocol. High Capacity cDNA Reverse Transcription kit (Life Technologies, Carlsbad, CA, USA) was used for the synthesis of cDNA, from 400 ng of total RNA from DRN extracts.

Quantitative real-time PCR was performed using Fast Start Universal SYBR Green Master (Rox; Roche) and ViiA™7 Real-Time PCR System (Life Technologies, Carlsbad, CA, USA). Relative quantification by ddCt method was used for analyses of gene expression. Primer sequences of analyzed genes: TPH2, serotonin autoreceptor (5-HT1A) and SERT, as well as housekeeping gene (actin; ACTB) are listed in the Supplementary Table S1.

### Statistical Analyses

Statistical analyses were done by SPSS. Data from GF and SPF mice were compared using two-tailed *t*-test. The effects of mice microbiological status and TRP depletion were analyzed by two-way analysis of variance (ANOVA), followed by LSD *post hoc* test for evaluating differences between specific groups. Values that fell out of the range mean ± 2 SD were considered as outliers and excluded from the analyses. The level of significance was set at *p* < 0.05. As a measure of the effect size of ATD in GF and SPF mice, *Cohen’s d* was used.

## Results

### GF Mice Show Less Depressive-Like Behavior Compared to SPF

**Figure 1:**
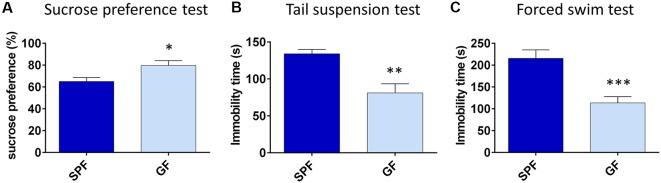
Germ free (GF) mice exhibit less depressive-like behavior than specific pathogen free (SPF) mice. GF mice have higher preference for 0.5% sucrose **(A)**, show less immobility time in tail suspension test **(B)** and forced swim test **(C)** compared to SPF mice. *p < 0.05, **p < 0.01, ***p < 0.001, two-tailed *t*-test, *N* = 10 mice per group.

First, we characterized the depressive-like behavior of GF mice compared to SPF. In the SPT, an established test of anhedonia, GF mice showed a greater preference for 0.5% sucrose compared to SPF (*t* = 2.34, *p* < 0.05, Figure 1A). Since we proposed that GF mice are hyperhedonic, we chose 0.5% sucrose according to our pilot experiment (Supplementary Figure S1), showing that SPF mice drink this percentage of sucrose solution significantly less than 1% or 2% solution of sucrose.

In tests of behavioral despair, GF mice were less immobile in both TST (*t* = −3.76, *p* = 0.001, Figure 1B) and FST (*t* = −4.40, *p* < 0.001, Figure 1C), in comparison to SPF. Altogether, GF mice exhibited less depressive-like behavior paralleled to their SPF counterparts.

### GF Mice Have Higher Expression of TPH2, SERT and 5-HTR1A in DRN

With the aim to obtain further insight into differences of serotonergic system of GF mice, and their depressive behavior, we assessed expression levels of TPH2, 5-HTR1A and SERT in DRN, being that it is the major source of serotonergic neurons projecting to the forebrain, involved in regulation of depressive behavior (Lesch and Waider, 2012).

**Figure 2:**
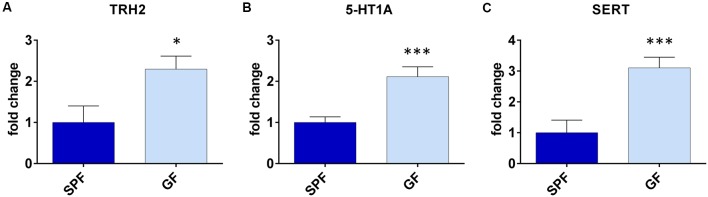
Germ free (GF) mice exhibit different gene expressions in dorsal raphe nucleus (DRN) compared to specific pathogen free (SPF) mice. GF mice have higher expression of tryptophan hydroxilase 2 (TPH2; **A**), serotonin autoreceptor 5-HT1A **(B)** and serotonin transporter (SERT; **C**) compared to SPF mice. *p < 0.05, ***p < 0.001, two-tailed *t*-test, *N* = 10 mice per group.

The expression of TPH2 was two times higher in GF mice compared to SPF (*t* = 2.50, *p* < 0.05, Figure 2A). Further, the expression of 5-HTR1A was three times higher (*t* = 4.16, *p* < 0.001, Figure 2B), while the SERT levels of mRNA were twice higher in GF mice than in SPF (*t* = 3.87, *p* < 0.01, Figure 2C). Therefore, the expression of genes regulating serotonin production and firing rate of serotonergic neurons, differed in GF mice, compared to SPF.

### GF Mice Are More Sensitive to TRP Depletion Than SPF

In the next set of experiments, we examined whether GF mice respond differently to the acute challenge of TRP deprivation compared to SPF, being that it is known their nervous system develops in conditions of high availability of TRP from their blood (Wikoff et al., 2009; Clarke et al., 2013). For that purpose, we performed ATD procedure.

#### Assessment of Depressive-Like Behavior

**Figure 3:**
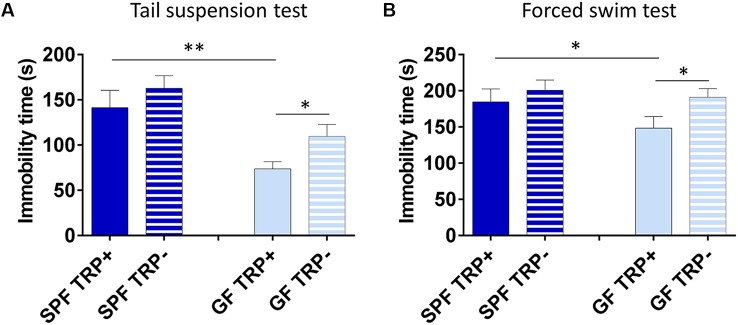
Effects of acute tryptophan depletion (ATD) on depressive-like behavior in germ free (GF) and specific pathogen free (SPF) mice. Changes in immobility time in tail suspension test **(A)** and forced swim test **(B)** after ATD in GF and SPF mice. **TRP**^+^ - group receiving control amino acid mixture with tryptophan, **TRP**^−^ - group receiving amino acid mixture without tryptophan. *p < 0.05, **p < 0.01, LSD *post hoc* test, *N* = 11 mice per group.

First, we determined how ATD affected depressive-like behavior of SPF and GF mice. In TST (Figure 3A), ANOVA showed a significant effect of both TRP depletion (*F* = 5.26, *p* < 0.05) and microbial status (*F* = 15.82, *p* < 0.001) of mice. However, SPF mice that underwent ATD did not significantly differ from mice receiving full amino acid mixture (*p* > 0.05, *Cohen’s d* = 0.41, Figure 3A). On the other hand, TRP depletion led to a strong increase of immobility in GF mice in comparison to control mixture (*p* < 0.05, *Cohen’s d* = 1.01, Figure 3A). Therefore, acute depletion of TRP from the diet had a stronger effect on GF mice than on SPF in TST.

Similarly, in FST (Figure 3B) the effect of ATD was significant (*F* = 4.94, *p* < 0.05), while the effect of microbial status was close to significant (*F* = 3.22, *p* = 0.08). Also, no significant differences were observed in SPF mice between TRP+ and TRP− groups (*p* > 0.05, *Cohen’s d* = 0.318, Figure 3B), while GF TRP− group showed increased immobility time compared to GF TRP+ group (*p* < 0.05, *Cohen’s d* = 0.94, Figure 3B). Therefore, acute depletion of TRP from diet had a stronger effect on GF mice than on SPF in FST as well.

Altogether, TRP depletion led to increased depressive-like behavior with larger effect size in GF mice compared to SPF.

#### Levels of TRP, 5-HT and 5-HIAA in mPFC

To gain insight into the mechanism of higher sensitivity of GF mice to ATD, we examined the levels of TRP, 5-HT and 5-HIAA in the brains of GF and SPF animals after ATD.

**Figure 4:**
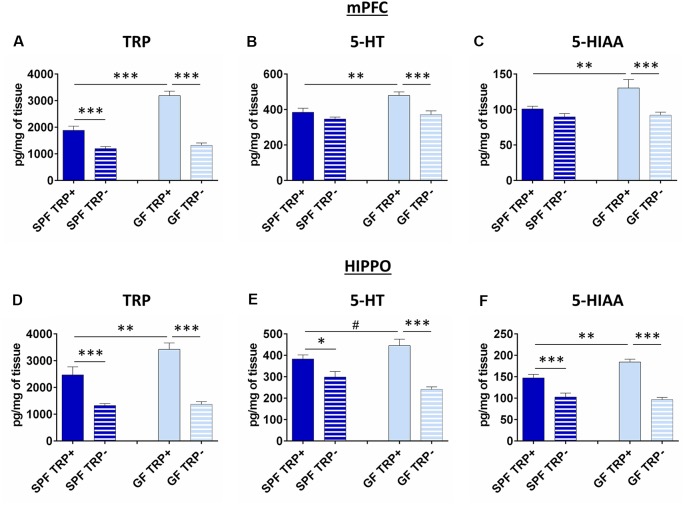
Effects of ATD on tryptophan, serotonin and its metabolite in the brain of GF and SPF mice. Changes in the levels of tryptophan (TRP; **A**), serotonin (5-HT; **B**) and the main serotonin metabolite (5-HIAA) in medial prefrontal cortex (mPFC) after ATD in GF and SPF mice. Changes in levels of TRP **(D)** serotonin **(E)** and 5-HIAA **(F)** in hippocampus (HIPPO) after ATD in GF and SPF mice. TRP^+^ - group receiving control amino acid mixture with tryptophan, TRP^−^ - group receiving amino acid mixture without tryptophan. ^#^1 > *p* > 0.05, *p < 0.05, **p < 0.01, ***p < 0.01, LSD *post hoc* test, *N* = 10 mice per group.

In mPFC, the brain region involved in coordination and control of depressive behavior (Drevets, 2000; Liu et al., 2017), levels of TRP were influenced by both TRP depletion (*F* = 99.32, *p* < 0.001) and GF state (*F* = 31.14, *p* < 0.001; Figure 4A). In mice not treated with TRP depletion, GF mice had higher TRP levels than SPF mice (*p* < 0.001, Figure 4A). In TRP depleted mice, TRP levels were significantly decreased, as expected. However, the effect of TRP depletion was much more pronounced in GF mice (*p* < 0.001, *Cohen’s d* = 4.47, Figure 4A) than in SPF mice (*p* < 0.001, *Cohen’s d* = 1.75, Figure 4A). Further, ANOVA showed a significant interaction between factors of ATD and microbial status (*F*_ATDxGF_ = 21.33, *p* < 0.001).

The levels of 5-HT (Figure 4B), as well as 5-HIAA (Figure 4C), which is the final product of 5-HT degradation, were also affected by both ATD (5-HT: *F* = 14.09, *p* < 0.001; 5-HIAA: *F* = 12.74, *p* = 0.001) and microbiota status (5-HT: *F* = 9.41, *p* < 0.01; 5-HIAA: *F* = 5.23, *p* < 0.05). In mice with no TRP depletion, GF mice had higher 5-HT and 5-HIAA levels compared to SPF (5-HT: *p* < 0.01, Figure 4B; 5-HIAA: *p* < 0.01, Figure 4C). After TRP depletion, levels of 5-HT and 5-HIAA were decreased only in GF mice (5-HT: *p* < 0.001, *Cohen’s d* = 1.65, Figure 4B; 5-HIAA: *p* < 0.001, *Cohen’s d* = 1.37, Figure 4C), and not in SPF mice (5-HT: *p* > 0.05, *Cohen’s d* = 0.32; 5-HIAA: *p* > 0.05, *Cohen’s d* = 0.84). The interactions of ATD and GF status at the levels of 5-HT and 5-HIAA were also close to significance (5-HT: *F*_ATDxGF_ = 3.16, *p* = 0.08; 5-HIAA: *F*_ATDxGF_ = 3.80, *p* = 0.06).

The ratio of 5-HT/5-HIAA, as a measure of 5-HT turnover, was not affected by any of the treatments (data not shown).

Therefore, GF mice had more 5-HT in the mPFC, but their 5-HT system showed higher vulnerability to ATD challenge, compared to SPF.

#### Levels of TRP, 5-HT and 5-HIAA in HIPPO

In HIPPO, a brain region also involved pathophysiology of depression (Campbell and Macqueen, 2004; Liu et al., 2017), the levels of TRP were affected by both TRP depletion (*F* = 64.48, *p* < 0.001) and GF status (*F* = 6.31, *p* < 0.05; Figure 4D), similarly to mPFC. In mice not subjected to TRP depletion, SPF group had lower levels of TRP compared to GF mice (*p* < 0.01, Figure 4D). ATD lead to TRP decrease in both SPF (*p* < 0.001, *Cohen’s d* = 1.66, Figure 4D) and GF mice (*p* < 0.001, *Cohen’s d* = 3.63, Figure 4D), as expected, but the effect was greater in GF animals. There was also a significant interaction of TRP depletion and microbiota status (*F*_ATDxGF_ = 5.16, *p* < 0.05).

Serotonin levels in HIPPO (Figure 4E) were affected by ATD treatment (*F* = 41.03, *p* < 0.001) but not by the absence of microbiota. There was a tendency of increased 5-HT levels in SPF mice compared to GF control mice (*p* = 0.058, Figure 4D). After TRP depletion, 5-HT levels dropped in both SPF (*p* < 0.05, *Cohen’s d* = 1.18, Figure 4D) and GF (*p* < 0.001, *Cohen’s d* = 2.88, Figure 4D) mice, but the effect was stronger in GF animals. Additionally, the interaction of TRP depletion and microbiota presence was significant (*F*_ATDxGF_ = 7.12, *p* < 0.05).

The levels of 5-HIAA (Figure 4F) were affected by both ATD procedure (*F* = 80.83, *p* < 0.001) and GF status (*F* = 4.84, *p* < 0.05). In mice not subjected to TRP depletion, GF had higher 5-HIAA levels compared to SPF (*p* < 0.01, Figure 4F). However, the decrease in 5-HIAA was higher in GF TRP− compared to GF TRP+ mice (*p* < 0.001, *Cohen’s d* = 4.55, Figure 4F), than in SPF TRP− compared to SPF TRP+ mice (*p* < 0.001, *Cohen’s d* = 1.60, Figure 4F). Also, the significant interaction of ATD and GF status was detected (*F*_ATDxGF_ = 8.99, *p* < 0.01).

There were no differences between groups in 5-HT turnover (data not shown).

In conclusion, 5-HT system in HIPPO, as in mPFC, was affected more strongly in GF mice than in SPF.

## Discussion

There is an increasing interest in dysbiosis of gut microbiota and its role in the development of various psychiatric problems, including depression. Our results show that GF mice exhibit less depressive-like phenotype, but they are more sensitive to acute TRP depletion challenge. These results give direct evidence that TRP availability to the brain and its subsequent regulation of serotonin levels is one mechanism through which gut microbiota may affect depression behavior.

We first characterized the depressive-like behavior of GF animals, compared to their counterparts with normal nonpathogenic commensal microbiota. Indeed, it was already reported that GF mice exhibit different behavior compared to control mice: they are less anxious, with altered social behavior, and impaired memory. Our results are in concert with previous data showing that GF mice display reduced immobility in tests of behavioral despair, TST (De Palma et al., 2015) and FST (Campos et al., 2016). However, contrary to recent data where the authors reported no difference in sucrose preference (Campos et al., 2016), our results showed that GF mice are also hyperhedonic compared to SPF mice. The reason for this discrepancy could be due to the fact that we used 0.5% of sucrose in the test of anhedonia, while Campos et al. (2016) used 2% sucrose solution for the test. Therefore, using a lower concentration of sucrose, we were able to detect an increased preference of GF mice for sucrose solution. Altogether, GF mice exhibit a strong reduction of depressive-like behavior.

To start revealing molecular mechanisms underlying these differences in depressive behavior, we evaluated levels of genes related to serotonin metabolism in DRN, the largest nucleus with serotonergic neurons projecting to the forebrain (Jacobs and Azmitia, 1992). We found increased levels of TPH2, the rate-limiting enzyme responsible for serotonin synthesis. The previous study of serotonin system in GF mice did not find differences in TPH2 in HIPPO (Clarke et al., 2013). However, since the DRN is the major site of serotonin synthesis, our results indicate that production of serotonin is increased in GF mice, as indeed was confirmed by HPLC analyses (discussed below). The expression of 5-HT1A autoreceptor and SERT were also increased in DRN of GF mice. In DRN, 5-HT1A is a somatodendric autoreceptor, a part of negative feedback loop, sensing the serotonin release and inhibiting further serotonin release, while SERT plays a role of serotonin clearance from the synaptic cleft (Jacobs and Azmitia, 1992). Therefore, an increased expression of these two components of serotonergic system in DRN could indicate that the firing rate of serotonin neurons is decreased. However, it could be also an adaptation mechanism to the high availability of TRP in GF animals, and consequently, high serotonin synthesis, leading to actually decreased depressive-like behavior in GF mice compared to SPF.

To further evaluate the role of microbiota in shaping serotonergic system and depressive behavior of the host organism, we used TRP depletion procedure, known to be a good indicator of vulnerability of brain serotonin system and predisposition to depression (Jans et al., 2007). In humans, TRP depletion induces a decrease in mood in depressive patients or healthy subjects with a family history of depression, but not in euthymic controls (Booij et al., 2003; Ruhé et al., 2007). Effects of ATD on anxiety and depression-like behavior in animal studies are mixed, likely reflecting variation in vulnerability of different rodent strains to serotonin system manipulation (Blokland et al., 2002; Jans et al., 2007, 2010; van Donkelaar et al., 2010; Biskup et al., 2012). In our study, GF mice, despite showing reduced basal levels of depression, were more sensitive to acute TRP reduction, i.e., exacerbation of their depression-like behavior was much stronger than in SPF mice. In fact, GF mice after TRP reduction displayed similar depressive-like behavior to SPF mice. The fact that ATD did not affect depressive-like behavior of control SPF Swiss Webster mice is in line with previous studies demonstrating that ATD did not change anxiety and depression-like behavior of SPF C57 mice (van Donkelaar et al., 2010) or Brown Norway rats (Jans et al., 2010). However, the same procedure augmented depressive-like behavior of GF mice. Therefore, our results indicate that the presence of microbiota can protect mice from the behavioral consequences of TRP depletion.

GF mice had higher TRP concentration in the brain than SPF mice, which is in accordance with previous data showing that GF mice have elevated plasma TRP levels (Wikoff et al., 2009; Clarke et al., 2013). Accordingly, levels of serotonin were also elevated, with a more pronounced increase in mPFC than in HIPPO. Indeed, expression of TPH2 in DRN, the major enzyme involved in the synthesis of brain serotonin, was two times higher in GF mice compared to SPF. The levels of 5-HIAA, a major metabolite of serotonin, was also increased in brains of GF mice receiving control amino acid mixture, while 5-HIAA/5-HT levels did not differ compared to SPF mice fed with control amino acid mixture. Therefore, it seems that in GF conditions, brain serotonin synthesis is increased, as so is its release and degradation, resulting in no change in its turnover. The higher levels of serotonin and 5-HIAA have already been reported in HIPPO of GF mice (Clarke et al., 2013). However, our data indicate that the elevation was more pronounced in mPFC than in HIPPO. Also, this increase of brain serotonin levels, especially in the mPFC, was parallel to the less depressive-like phenotype of GF mice.

After TRP depletion, the decrease of brain TRP was much more pronounced in GF mice than in SPF. This TRP reduction resulted in decreased serotonin levels in mPFC of GF mice but not SPF mice. In HIPPO, after ATD, serotonin levels were lower in both GF and SPF mice, but the decrease was more prominent in GF mice. Therefore, the stronger effect of TRP depletion on brain serotonin reduction in GF mice was in concordance with their increased depressive-like behavior after ATD.

There are several possible explanations of why SPF mice are less vulnerable to ATD, both at the molecular and behavioral levels, compared to GF mice. First, microbiota induces the production of serotonin from TRP in gut enterochromaffin cells (Yano et al., 2015), therefore decreasing the availability of dietary TRP to enter the bloodstream and brain, and to be converted to serotonin in the brain. In GF animals, in contrast, the ingested TRP will be more readily available to enter the brain, induce serotonin production, and have behavioral effects. Therefore, the action of enterochromaffin cells may attenuate the effects of ATD in SPF mice. In other words, in such way, SPF mice can be less vulnerable to the acute effects of dietary fluctuation in TRP levels. Second, there is also a possibility that TRP and serotonin produced by gut bacteria (Priya et al., 2014; Roshchina, 2016) may be supplementing the brain of SPF mice, therefore attenuating the effect of ATD. However, this is a more controversial claim. Namely, although there are some data showing that bacteria can produce TRP (Priya et al., 2014), it is still regarded that the main source of TRP for mammals is supplied by ingested food (Agus et al., 2018). Then, it is considered that most brain serotonin is produced in specific neurons of the brainstem and it does not cross the blood brain barrier (Ruddick et al., 2006; El-Merahbi et al., 2015). However, there is some evidence that brain endothelial cells express SERT, opening a possibility that blood can provide the brain with serotonin (Brust et al., 2000; Wakayama et al., 2002) which could certainly be an intriguing topic for further research. Finally, microbiota can be an important factor in the shaping of brain serotonergic system as also observed in our data by increased expression of TPH2, 5-HT1A and SERT in DRN of GF mice (Clarke et al., 2013; O’Mahony et al., 2015), and in such way it can possibly influence on the sensitivity of the serotonergic system to acute TRP deficits as well.

Overall, we have found that the presence of microbiota in mice is accompanied by lower serotonin levels and higher levels of depressive-like behavior. In contrast, the presence of microbiota can provide a better resistance to mood lowering effect induced by acute depletion of brain TRP. Therefore, this suggests that microbiota regulation of depressive behavior is complex, and may be disadvantageous or beneficial, dependent on the environmental conditions (i.e., baseline or conditions of reduced TRP brain levels), which is also in line with previous data. For example, it was shown that GF mice, despite exhibiting anxiolytic and anti-depressive phenotype, have more exaggerated rise of corticosterone, in a response to restrained stress (Sudo et al., 2004; Diaz Heijtz et al., 2011; Neufeld et al., 2011; Clarke et al., 2013), which is related to increased predisposition for depression (de Kloet et al., 2005). On the other hand, GF mice showed hypo-responsiveness to LPS challenge as well as maternal separation (De Palma et al., 2015; Campos et al., 2016). Namely, in the absence of microbiota, mice were resistant to LPS-induced depressive-like changes in behavior (Campos et al., 2016). In a separate study, examining the effects of early life stress, gut microbiota was shown to be an important factor driving depressive and anxiety-like phenotype later in the adult life, while GF mice were resilient to such experience (De Palma et al., 2015). Therefore, normal gut microbiota could be protective to some vulnerability factors towards developing depression, while mediating unfavorable effects of some other depression vulnerability factors. Which particular bacterial groups are involved in these effects is an avenue for future research.

In conclusion, our data revealed anti-depressive effect of microbiota absence, but also higher sensitivity of GF mice in response to the challenge of TRP depletion. These results suggest that microbiota, through affecting brain TRP availability, affects the brain serotonergic system of the host, and can influence the vulnerability to depression.

## Ethics Statement

The study was carried out in accordance with the recommendations of the Institutional Animal Care and Use Committee of Bar Ilan University Faculty of Medicine and the protocol for this project was approved by the Institutional Animal Care and Use Committee of Bar Ilan University Faculty of Medicine.

## Author Contributions

IL took part in designing the study, conducted experimental work, analyzed the data, and wrote the manuscript. DG helped in conducting the experimental work. OK took part in analyzing the results and revised the manuscript. EE designed the study, discussed results and revised the manuscript.

## Conflict of Interest Statement

The authors declare that the research was conducted in the absence of any commercial or financial relationships that could be construed as a potential conflict of interest.

## Abbreviations

ATD: acute tryptophan depletion
DRN: dorsal raphe nucleus
FST: forced swim test
GF: germ free
HIPPO: hippocampus
mPFC: medial prefrontal cortex
SPF: specific pathogen free
TRP: tryptophan
TST: tail suspension test
5-HT: 5-hydroxytryptamine
5-HIAA: 5-hydroxyindoleacetic acid
SERT: serotonin transporter
TPH2: tryptophan hydroxylase 2.
