# Supplementary material for: Role of Tryptophan in Microbiota-Induced Depressive-Like Behavior: Evidence From Tryptophan Depletion Study
Source: Front Behav Neurosci. 2019 Jun 4;13:123. doi: 10.3389/fnbeh.2019.00123 (PMC6558209; doi:10.3389/fnbeh.2019.00123)
Supplement: Supplementary file 1 [file Data_Sheet_1.docx]

**Role Of Tryptophan In Microbiota-Induced Depressive-Like Behavior: Evidence From Tryptophan Depletion Study**

Iva Lukić, Dmitriy Getselter, Omry Koren and Evan Elliott

**Supplementary Material**

**
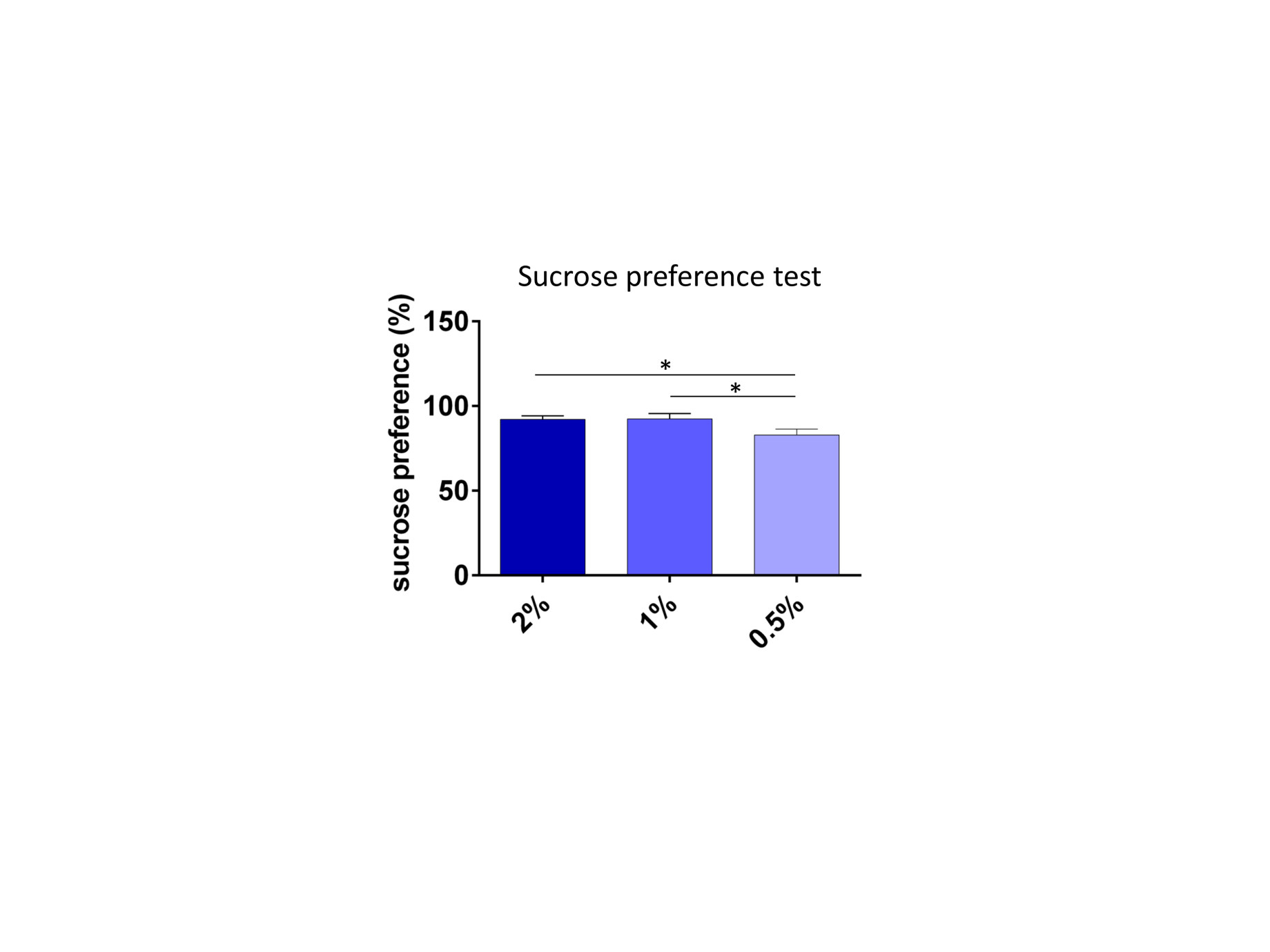
**

**Supplementary Figure S1.** Pilot experiment of sucrose preference test with different concentrations of sucrose solution (2%, 1% and 0.5%). The experiment was done in SPF Swiss Webster mice. ANOVA: F=3.50, p=0.063; *p<0.05, LSD post hoc test

**Supplementary Table S1.** Sequences of primers used in the study.

| **Reverse primer** | **Forward primer** | **Name of the target gene** |
| --- | --- | --- |
| CTCGTTGCCAATAGTGATGACCTG | CCACTGCCGCATCCTCTTCC | ACTB |
| AAGCCAGATCGCTCTTTCAGA | TCTACCCGACTCATGCTTGC | TPH2 |
| GGCTGACCATTCAGGCTCTT | CATGCTGGTCCTCTATGGGC | HTR1A |
| ACACCCCTGTCTCCAAGAGT | CAAAACGTCTGGCAAGGTGG | SERT |
